# The roles of ncRNAs and histone-modifiers in regulating breast cancer stem cells

**DOI:** 10.1007/s13238-015-0199-4

**Published:** 2015-09-08

**Authors:** Zhiju Zhao, Shu Li, Erwei Song, Suling Liu

**Affiliations:** Innovation Center for Cell Signalling and the CAS Key Laboratory of Innate Immunity and Chronic Disease, School of Life Sciences and Medical Center, University of Science & Technology of China, Hefei, 230027 China; Department of Pathophysiology, Wannan Medical College, Wuhu, 241002 China; Department of Breast Surgery, Sun Yat-sen Memorial Hospital, Sun Yat-Sen University, Guangzhou, 510120 China

**Keywords:** breast cancer stem cells, microRNA, lncRNA, histone-modifier, Polycomb group proteins

## Abstract

Cancer stem cells (CSCs), a subpopulation of cancer cells with ability of initiating tumorigenesis, exist in many kinds of tumors including breast cancer. Cancer stem cells contribute to treatment resistance and relapse. Conventional treatments only kill differentiated cancer cells, but spare CSCs. Combining conventional treatments with therapeutic drugs targeting to CSCs will eradicate cancer cells more efficiently. Studying the molecular mechanisms of CSCs regulation is essential for developing new therapeutic strategies. Growing evidences showed CSCs are regulated by non-coding RNA (ncRNA) including microRNAs and long non-coding RNAs (lncRNAs), and histone-modifiers, such as let-7, miR-93, miR-100, HOTAIR, Bmi-1 and EZH2. Herein we review the roles of microRNAs, lncRNAs and histone-modifiers especially Polycomb family proteins in regulating breast cancer stem cells (BCSCs).

## INTRODUCTION

The concept of cancer stem cells (CSCs) has been generally accepted since leukaemia cancer stem cells were discovered by John E. Dick in 1994 (Lapidot et al., 1994). CSCs maintain CSCs pool or generate more CSCs via self-renewal, and generate non-CSCs progenies forming the differentiated cancer cells via differentiation. CSCs also initiate tumor formation, required critical cell amount for tumor formation in xenografts is reduced compared to non-CSCs. It has been reported that CSCs exist in many kinds of tumors, including breast cancer, lung cancer, leukaemia, glioblastoma, colon cancer, live cancer and so on (Visvader and Lindeman, 2008), meanwhile, some molecular markers have been used to separate CSCs from total cancer cell population, such as ALDH (Ginestier et al., 2007) and CD24^−^CD44^+^ (Al-Hajj et al., 2003) for BCSCs, CD90 (Yang et al., 2008) and CD133 (Ma et al., 2007) for liver cancer stem cells, ABCB5 and CD271^+^ for melanoma cancer stem cells (Schatton et al., 2008), CD133 for brain tumor stem cells (Singh et al., 2004). CSCs are heterogenous, for example, BCSCs and colon cancer stem cells include at least two types of CSCs identified with different molecular markers (Liu et al., 2014). BCR-ABL1 lymphoblastic leukaemia contains multiple genetically distinct leukaemia stem cell sub-clones (Notta et al., 2011).

It has revealed that some signaling pathways play critical roles in regulating the self-renewal and differentiation of CSCs. Wnt signaling is important for CSC self-renewal, for example, Wnt/β-catenin signaling is activated in poor differentiated basal-like breast cancer with worse overall survival (Khramtsov et al., 2010). Constitutive activation of Wnt signaling caused by the mutation of tumor suppressor APC leads to breast cancer stem cell (BCSC) expansion. In Her2^+^ breast cancer inhibition of Wnt signaling represses tumor initiation and metastasis (Monteiro et al., 2014; Schade et al., 2013). These suggest deregulated Wnt signaling promotes the expansion of CSCs. Hedgehog (HH) signaling promotes glioma growth by stimulating self-renewal of CD133^+^ glioma CSCs, and increases chemotherapeutic agent resistance (Clement et al., 2007). Hedgehog signaling also maintain CSCs in breast cancer and myeloid leukaemia (Liu et al., 2006; Zhao et al., 2009). Notch signaling is also important in regulating CSCs (Pannuti et al., 2010). For example, Notch-GFP reporter system has been used to separate CSCs from lung adenocarcinoma and breast cancer, GFP^+^ cancer cells could differentiate into GFP^−^ cancer cells, and have strong tumor initiation capacity (D’Angelo et al., 2015; Hassan et al., 2013). Notch signaling induces deacetylase sirtuin 2 (SIRT2) to deacetylate and activate ALDH1A1 and then increases BCSCs (Zhao et al., 2014). Apart from these signaling pathways, TGF-β, IL6/JAK/STAT3, NF-κB signaling and other signaling pathways also play critical role in regulating CSCs, and they sometimes cross-talk with each other in the regulation.

With the development of epigenetics, histone-modifying enzymes and ncRNAs have been found to play vital roles in regulating CSCs. In this review, we focus on the research progress about ncRNAs and histone-modifiers in regulating BCSCs.

## BREAST CANCER STEM CELLS

In 2003, Clarke and his colleagues isolated putative BCSCs as ESA^+^CD44^+^CD24^−^ cell population (Al-Hajj et al., 2003). As few as 200 ESA^+^CD44^+^CD24^−^ cells were capable to generate tumor *in vivo*, whereas a 100-fold more cells without these markers isolated from the same tumors were non-tumorigenic. In addition, the secondary tumors resemble the phenotype (morphology and ESA/CD44/CD24 expression profile) of the initial tumor and the tumorigenic ESA^+^CD44^+^CD24^−^ tumor cells could be serially passaged at least four passages *in vivo*. Subsequent studies employed several methodologies adapted from stem cell research to isolate or investigate BCSCs, including side population (SP) assay, ALDEFLUOR assay and sphere assay. The SP assay is based on the ability of stem cells to exclude DNA dye such as Hoechst 33342 by membrane transporters, and the SP has been shown to contain the most tumorigenic population within breast cancer cell line when being transplanted *in vivo* (Dontu et al., 2003). The Aldefluor assay represents a group of enzymes catalyzing the oxidation of aldehydes. In malignant mammary epithelium, cells with high Aldehyde dehydrogenase (ALDH) activity were associated with the greatest self-renewal and differentiation abilities both *in vitro* and *in vivo*, and positive ALDH immunostaining in breast carcinomas correlated with poor prognosis (Ginestier et al., 2007). Mammary stem/progenitor cells are able to survive in serum-free and anchorage-independent conditions in the form of spheroids (Dontu et al., 2003). BCSCs were also enriched when grown as non-adherent spheroids *in vitro* (Ponti et al., 2005). Interestingly, the two tumor initiating populations (ALDH^+^ cells and ESA^+^CD44^+^CD24^−^ cells) only showed limited overlapping (Box 1) (Ginestier and Wicha, 2007). Similar finding was also demonstrated by the other group that breast cancers may contain tumor initiating cells displaying different cell surface markers (Wright et al., 2008). Despite the heterogeneity of BCSCs, these cells are usually associated with therapy resistance and tumor relapse, the two main obstacles in cancer treatment. Therefore, understanding the biology of CSCs will help the development of new therapeutic approaches to target CSCs, leading to more effective therapies and ultimate cure for cancer.

**Box 1:** Different types of breast cancer stem cells

ALDH^+^ and CD24^−^CD44^+^ are different markers for breast cancer stem cells (BCSCs) (Liu et al., 2014). CD24^−^CD44^+^ marks BCSCs in a mesenchymal-like (EMT) state, primarily quiescent, and localized at the tumor invasion front; ALDH^+^ marked BCSCs with epithelial-like (MET) state, proliferative, and localized at the tumor center. They both can self-renew and differentiate. The tumorigenesis ability of BCSCs in the overlap of ALDH^+^ and CD24^−^CD44^+^ is the highest. These two types of BCSCs can reciprocally transform, which could be induced by tumor microenvironmental factors, microRNAs, lncRNAs or epigenetic proteins.	

## BREAST CANCER STEM CELLS (BCSCs) ARE REGULATED BY microRNAs

MicroRNAs (miRNAs) regulate targeted mRNAs through a combination of translational repression and mRNA destabilization. The biogenesis of miRNAs has been summarized in details by V. Narry Kim (Kim et al., 2009). Studies have shown microRNAs regulate cells proliferation, invasion, metastasis and angiogenesis in both solid tumors and leukemia (Nicoloso et al., 2009). miR-29 promotes hepatocellular carcinoma cell apoptosis by targeting Mcl-1 and Bcl-2 (Xiong et al., 2010). miR-10b initiates tumor invasion and metastasis by targeting RHOC in breast cancer (Ma et al., 2008).

In recent years, miRNAs have been studied in BCSCs intensively (Table 1). We have shown that let-7a is downregulated in mammosperes in comparison to differentiated cancer cells utilizing miRNA array analysis; let-7a is also lower in BCSCs marked by CD24^−^CD44^+^ than non-CD24^−^CD44^+^ cells, and let-7a overexpression suppressed the mammosphere formation and tumor initiation. Further analysis reveals let-7a suppresses self-renewal of BCSCs in part by targeting H-Ras, and promotes cellular differentiation by targeting HMGA2 (Yu et al., 2007). Let-7 is also regulated by some signaling pathways, e.g., Wnt-β-catenin pathway activates Lin28 which suppress let-7 biogenesis by inducing urdylation of precursor let-7 (pre-let-7) at its 3′ end and then represses let-7 to expand CSCs (Cai et al., 2013; Heo et al., 2008). Some protein methyltransferases not only catalyze methylation of histones, but also nonhistone proteins. For example, SET7/9 which catalyzes monomethylation of histone 3 also catalyzes methylation of Lin28A at K135 to promote Lin28 accumulation in nucleus, and increases the stability and pri-let-7-binding ability of Lin28 (Kim et al., 2014), suggesting epigenetic proteins can regulate CSCs.

**Table 1:** **miRNAs aberrantly expressed and validated target genes in BCSCs**

miRNA	Expression	Targets	Function	References
let-7	Down	H-Ras, HMGA2	Reduce BCSCs	Yu et al. (2007)
miR-200c	Down	BMI1	Reduce BCSCs	Shimono et al. (2009)
miR-200b	Down	SUZ12	Reduce BCSCs	Iliopoulos et al. (2010)
miR-93	Down	AKT3, SOX4 and STAT3	Inhibit BCSCs in basal type cancer, promote BCSCs in luminal type cancer	Liu et al. (2012)
miR-100	Down	SMARCA5, SMARCD1 and BMPR2	Inhibit BCSCs	Deng et al., (2014)
miR-141	Down	Stat5a and PR	Reduce BCSCs	Finlay-Schultz et al. (2014)
miR-34c	Down	Notch4	Inhibit BCSCs	Yu et al. (2012)
miR-30	Down	Ubc9 and ITGB3	Inhibit BCSCs	Yu et al. (2010)
miR-128	Down	BMI1 and ABCC5	Inhibit BCSCs	Zhu et al. (2011)
miR-140	Down	ALDH1 and SOX9	Inhibit BCSCs	Zhang et al. (2012)
miR-27a	Down	ZBTB10	Inhibit BCSCs	Tang et al. (2014)
miR-27b	Down	ENPP1	Inhibit BCSCS	Takahashi et al. (2015)
miR-7	Down	KLF4, SETDB1	Inhibit BCSCs	Okuda et al. (2013); Zhang et al. (2014a)
miR-34a	Down	Notch1	Reduce BCSCs	Park et al. (2014)
miR-142	Up	APC	Promote BCSCs	Isobe et al. (2014)
miR-21	Up	PTEN, AKT and ERK1/2 pathways	promote BCSCs	Han et al. (2012a, b, c)
miR-29	Up	Non report	Promote BCSCs	Li et al. (2014)
miR-495	Up	E-Cadherin	Promote BCSCs	Hwang-Verslues et al. (2011)
miR-181	Up	ATM	Promote BCSCs	Finlay-Schultz et al. (2014)
miR-22	Up	TET-family proteins	Promote BCSCs	Song et al. (2013)
miR-221	Up	ATXN1	Promote BCSCs	Ke et al. (2015)
miR-9	Up	Non-report	Promote BCSCs	Gwak et al. (2014)

**Figure 1:**
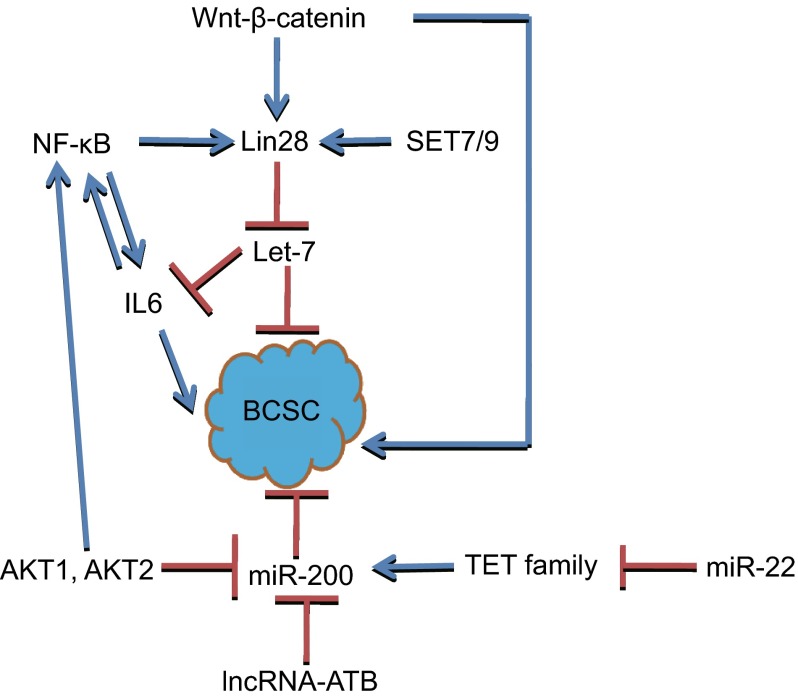
**Let-7 and miR-200 inhibit BCSCs**. Wnt-β-catenin could regulate BCSCs not only by regulating Lin28, but also by other proteins. Lin28 can be activated by NF-κB and SET7/9. Lin28 inhibits let-7. AKT1 and AKT2 suppress miR-200, and also activate NF-κB to regulate BCSCs. miR-22 inhibits TET family proteins which can activate miR-200, and lncRNA-ATB also inhibits miR-200

miR-200 family, including miR-200a, miR-200b, miR-200c, miR-141 and miR-429, are reported as tumor suppressors. They repress EMT by targeting ZEB1 and ZEB2. miR-200c suppresses BCSCs through targeting BMI1 (Shimono et al., 2009). miR-200b inhibits BCSCs by targeting SUZ12, H3K27me3 of E-cadherin and other genes (Iliopoulos et al., 2010). Inhibition of miR-141 increases both CD44^+^ and CK5^+^ cells by targeting Stat5a and progesterone receptor (PR), and enhances the abilities of mammosphere formation and tumor initiation. miR-200 family could be regulated by some signaling pathways, for example, abnormal expression of AKT1 and AKT2 causes dysregulation of miR-200 family to regulate epithelial-mesenchymal transition (EMT) and CSCs self-renewal (Iliopoulos et al., 2009). Besides ZEB1 and ZEB2, some Polycomb group (PcG) proteins are also targets of miR-200. The role of some Polycomb family proteins will be introduced later in the text (Fig. 1).

Recently, Pier Paolo Pandolfi and colleagues found miR-22 promotes the EMT, tumor invasion and metastasis of normal and cancer mammary stem cells by targeting TET1, TET2 and TET3; miR-22 overexpression enhances some stemness and EMT-related genes expression, such as BMI1, ZEB1 and ZEB2. TET1, TET2 and TET3 could inhibit the demethylation of miR-200 promoter. So miR-22 promotes BCSCs by suppressing miR-200 expression, suggesting DNA demethylases could regulate BCSCs.

miR-93 plays different roles in BCSCs come from different subtypes of breast cancer. miR-93 inhibits CSCs and initiates mesenchymal-epithelial transition (MET) in basal type of breast cancer cells such as SUM159 by targeting AKT3, SOX4 and STAT3. However, it promotes BCSCs in luminal type of breast cancer such as MCF-7, suggesting the dual roles of miR-93 in regulating BCSCs is dependent on the state of cellular differentiation, but the mechanism is yet to be elucidated (Liu et al., 2012).

In addition to BCSCs, dysregulation of miRNAs is also found in other types of CSCs. In colon cancer stem cells, the function of miR-34a depends on its expression levels: High miR-34a expression suppresses Notch signaling pathway and promotes differentiation of CSCs; low miR-34a expression promotes Notch signaling pathway and maintains CSCs phenotype (Bu et al., 2013). CD44 is a marker of prostate cancer stem cells, miR-34a inhibits CSCs and cancer metastasis by targeting CD44 (Liu et al., 2011). miR-218 inhibits glioma cancer stem cells by targeting BMI1 (Tu et al., 2013). More and more studies showed that miRNAs can potentially be used for tumor therapy by being linked to therapeutic vectors, such as nanoparticles.

## lncRNAs PLAY POTENTIAL ROLES IN REGULATING BREAST CANCER AND CANCER STEM CELLS

lncRNAs (long non-coding RNAs) are >200 nt non-coding RNA. A great number of lncRNAs have been discovered, but only a few lncRNAs have been well studied by now. Recently, lncRNAs have been studied in many cancers. For example, lncRNA-ATB activated by TGF-β induces EMT in hepatocellular carcinoma, breast cancer and colon cancer (Yuan et al., 2014). It not only function as a competing endogeneous RNA (ceRNA) competitively binding to miR-200 family to upregulate their targets and induces EMT, but also binds to IL11 mRNA, increasing the stability of IL11 and causing autocrine induction of IL11 to activate STAT3 pathway, which plays a vital role in regulating BCSCs. lncRNA-ATB may regulate BCSCs by regulating miR-200 family and STAT3.

Chemokine CCL21 binds to its receptor CCR7 to induce the phosphorylation of GLI2 mediated by citron (CIT) kinase, phosphorylation of GLI2 activates the target genes of GLI. lncRNA *BCAR4* is required for transcription activation of GLI2 target genes. RNA pull-down and mass spectrometry analysis reveals that *BCAR4* interacts with SNIP1 and PNUTS. When *BCAR4* interacts with SNIP1, SNIP1 releases the suppression of p300, and p300 acetylates GLI2 target gene promoters marked H3K18ac and promotes gene transcription. The acetylated H3K18 can be recognized by PNUTS, and interact with it to activate the phosphatase activity of PP1 to maintain hypophosphorylation level of RNA Pol II Ser5 at gene promoter regions. *BCAR4* could induce the activation of GLI’s target genes and promotes breast cancer metastasis, especially triple-negative breast cancer (Xing et al., 2014). The target genes of GLI plays pivotal role in BCSCs. These suggest that *BCAR4* may regulate BCSCs, which is to be demonstrated with further studies.

Furthermore, lncRNA *lncTCF7* regulated self-renewal of hepatocellular carcinoma stem cells demonstrated by tumorsphere formation ability *in vitro* and tumor initiating frequency *in vivo*. *lncTCF7* recruits the SWI/SNF complex to bind to TCF7 promoter and activate TCF7 expression, and TCF7 activates Wnt pathway to expand hepatocellular carcinoma stem cells (Wang et al., 2015). lncRNA-ROR is a modulator of cell reprogramming and pluripotency. In breast cancer, lncRNA-ROR induces EMT and promotes metastasis, lncRNA-ROR overexpression increases the percentage of CD24^−^CD44^+^ cell population and mammosphere numbers. Further analysis reveals that it can act as a ceRNA of miR-205 which targets the EMT inducer ZEB2 and blocks the degradation of ZEB2 to promote EMT (Hou et al., 2014).

HOTAIR has been studied for many types of cancers (Zhang et al., 2014b). In breast cancer HOTAIR promotes cancer metastasis (Gupta et al., 2010). It can act as a scaffold to bring two epigenetic protein complexes. The 5′ domain of HOTAIR binds to Ploycomb repressive complex 2 (PRC2), and the 3′ domian binds to the LSD1/CoREST/REST complex. HOTAIR can regulate the function of epigenetic complex and causes chromatin state change (Tsai et al., 2010). HOTAIR also regulates BCSCs, and microarray analysis reveals HOTAIR overexpression upregualtes the genes related to stemness and EMT, such as CD44, STAT3, ALDH2, ZEB1 and VIM, but the tumor initiating frequency *in vivo* assay are needed to demonstrate the role of HOTAIR in regulating BCSCs further (Padua Alves et al., 2013).

With the progression of studies about lncRNAs, more and more lncRNAs will been demonstrated in modulating CSCs. lncRNAs represent a new type of CSCs regulator by regulating miRNAs, mRNAs and other lncRNAs. The study of lncRNAs will improve the understanding of novel molecular regulation of CSCs, and lncRNAs can function as targets for novel therapies and as prognosis factors.

## HISTONE-MODIFIERS, ANOTHER VITAL REGULATOR OF BREAST CANCER STEM CELLS (BCSCs)

Histone H2A, H2B, H3 and H4 is modified by histone modifying enzymes, including histone acetyltransferases, histone deacetylases, histone methyltransferase and histone demethylases. Histone modifying enzymes play a role in the regulation of transcription by modulating the state of chromatin, and they also cross-talk with each other (Portela and Esteller, 2010). Studies about the roles of histone modifying enzymes in breast cancer and other cancers have been reported (Patani et al., 2011). H3K4 demethylase Jarid1B/KDM5B is amplified and overexpressed in breast cancer to promote cell proliferation. RNA-seq and ChIP-seq analysis revealed the binding sites of Jarid1B are significantly enriched in the promoters and enhancers of luminal-high genes than those of basal-high genes, indicating it is a luminal lineage-driving oncogene (Yamamoto et al., 2014). H3K9me2 methyltransferase G9a interacts with Snail and DNA methyltransferase, recruits them to the promoter of E-cadherin for DNA methylation and promotes EMT in breast cancer (Dong et al., 2012). Coactivator-associated arginine methyltransferase 1 (CARM1) methylates BAF155 at R1064. Methylation of BAF155 promotes breast cancer invasion and metastasis *in vivo* and *in vitro*. Chromatin immunoprecipitation (ChIP) analysis demonstrates CARM1 control the expression of genes in the c-myc pathway (Wang et al., 2014).

## NYD1/KDM2B

H3K36me1/2 and H3K4me3 demethylase NYD1/KDM2B plays an important role in ES and iPS. It is directly regulated by pluripotency factors OCT4 and SOX2, and it interacts with the core subunits of Ploycomb repressive complex 1 (PRC1), such as Ring1B and recruits PRC1 to the CpG island of promoter of genes which control the differentiation, resulting in the inhibition of differentiation genes. This also suggests KDM2B can function as a Polycomb group repressive element (PRE) to inhibit differentiation gene expression (He et al., 2013). KDM2B also inhibits let-7 and miR-101 to induce upregulation of EZH2 and the levels of H3K27me3 in the sites of *Ink4a-Arf-Ink4b*. So KDM2B can function as an oncogene (Kottakis et al., 2011; Tzatsos et al., 2011). In breast cancer, downreguation of KDM2B inhibits anchorage-dependent and -independent growth, arrests cell cycle in G_1_ and promotes apoptosis, and CSCs were also reduced. These results suggest KDM2B is an oncogene, not only promoting breast cancer but also maintaining BCSCs. Basal marker and luminal marker analysis showed KDM2B is required for the maintenance of the myoepithelial/luminal progenitor cell phenotype of basal breast cancer cells. Western blot analysis indicates Polycomb group (PcG) proteins, SUZ12, EZH2, RING1B and BMI1, are downregulated upon KDM2B knockdown, but they are not the direct downstream of KDM2B. KDM2B binds to the sites encoding miR-200 family, miR-101, miR-181 and miR-203. SUZ12, EZH2, RING1B and BMI1 are the direct targets of these miRNAs, and KDM2B inhibits BCSCs through repressing these miRNAs and upregulation of the core subunits of PcG proteins (Kottakis et al., 2014). In clinical samples, KDM2B expression has a negative correlation with these miRNAs, but has a positive correlation with these core subunits of PcG proteins. So PcG proteins have prominent role in BCSCs. RING1B is an E3 ubiquitin ligase for H2AK119, but the role of RING1B in breast stem cells have to be studied further in BCSCs.

PcG proteins are divided into two main subfamilies of complexes: PRC1 and PRC2. The targets of PcG proteins are highly enriched for transcription factors of signaling pathways involved in development and disease. With the new algorithms emerging, more targets of PcG proteins will be identified, including lncRNAs and miRNAs. The core subunits of PRC2 catalyze H3K27me3 in target sites, and H3K27me3 recruits PRC1 to inhibit target gene expression (Kerppola, 2009; Simon and Kingston, 2009). In addition to this mechanism, RYBP1-PRC1 complexes mediate H2A ubiquitylation at the Polycomb target sites also suppress the expression of targets independent on PRC2 and H3K27me3(Tavares et al., 2012).

## EZH2 and SUZ12

EZH2 and SUZ12 belong to PRC2. EZH2 is a histone methyltransferase and catalyzes H3K27me3, and SUZ12 stimulates the activity of EZH2. EZH2 plays critical roles in embryonic stem cells, adult stem cells and cancer. For example, in mammary gland, EZH2 maintains luminal progenitor cell self-renewal (Michalak et al., 2013). EZH2 is also an oncogene and the downstream of the pRB-E2F pathway. EZH2 is essential for proliferation and amplified in many primary cancers, and is inhibited by AKT through phosphorylation at Ser^21^ (Cha et al., 2005). EZH2 can be used as a molecular marker for precancerous diagnosis, and EZH2 overexpression in histologically normal breast epithelium increases the risk of developing cancer. Furthermore, EZH2 is upregulated in breast cancer, and high EZH2 levels are associated with aggressive breast cancer. Kaplan-Meier analysis of metastasis-free survival and overall survival show the survival rate is lower in patients with high EZH2 (Kleer et al., 2003). It has been well-known that hypoxia promotes CSCs, such as glioma stem cells (Li et al., 2009), colorectal cancer stem cells (Yeung et al., 2011) and BCSCs (Conley et al., 2012). Chun-Ju Chang and colleagues found hypoxia induces the expression of EZH2 to expand BCSCs, and EZH2 overexpression increases the number of SP and CD24^−^CD44^+^ cells. But the mechanism that hypoxia regulating EZH2 has to be elucidated. EZH2 inhibits the expression of tumor suppressor RAD51 which participates in DNA repair leading to genomic instability and increases some oncogene expression such as RAF1 which activates ERK and Wnt-β-catenin pathway to promote cancer cell survival and proliferation, and expands BCSCs (Chang et al., 2011). Notch signaling pathway maintains the stemness of cancer stem cells, induces EMT transition and promotes chemoresistance (Pannuti et al., 2010). In clinical samples, the expression of EZH2 and Notch1 are positively correlated. EZH2 knockdown inhibits the onset and growth of xenografts derived from triple-negative breast cancer, and the opposite phenotype emerges when EZH2 is overexpressed. EZH2 overexpression activates Notch1 signaling activity to promote BCSC self-renewal. Further analysis indicates EZH2 regulates Notch transcriptional activity depending on direct binding to the promoter of Notch1 rather than its histone methyltransferase activity. In glioblastoma stem cells, EZH2 activates STAT3 signaling and promotes tumorigenicity (Kim et al., 2013). Activation of STAT3 also promotes BCSCs, and intronic RNAs also mediate regulation of epigenetic targets (Guil et al., 2012). Recently, Hae-Yun Jung and colleagues find PAF (PCNA-associated factor) interacts with β-catenin to recruit EZH2 and form a transcriptional complex, and this complex specifically transactivates the target genes of Wnt signaling, suggesting EZH2 expands BCSCs by activating Wnt pathway (Jung et al., 2013), which indicates EZH2 may be a central protein, and is regulated by several key signaling pathways which regulate CSCs. Further studies are needed to reveal the regulatory mechanism.

SUZ12 promotes the silencing of Hox gene, cell proliferation and embryogenesis (Cao and Zhang, 2004; Pasini et al., 2004). The mutations of SUZ12 often are found in some cancer, for example, SUZ12 mutations cause the malignant transformation of peripheral nerve sheath tumors (Zhang et al., 2014c). SUZ12 knockdown inhibits mammosphere formation ability of BCSCs, and suppresses CD44. SUZ12 is also a direct target miR-200b which is a BCSC suppressor. miR-200b inhibits BCSCs through SUZ12 partly, but the molecular mechanism of SUZ12 regulating BCSCs is yet to be elucidated.

## BMI1

**Figure 2:**
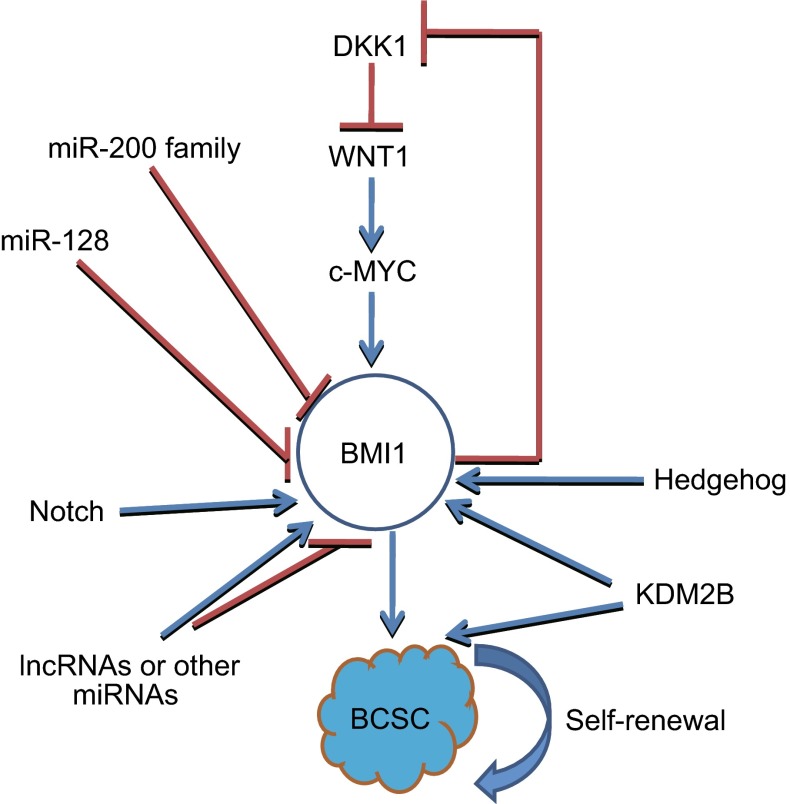
**BMI1 regulates BCSC**. BMI1 is activated by Hedgehog, Notch and Wnt pathway, and it is inactivated by miR-200 family and miR-128. There may be some other microRNAs and lncRNAs regulated BMI1

BMI1 which a canonical component of PRC1, is a co-factor for E3 ubiquitin ligase and compact polynucleosomes. Growing evidences suggest BMI1 plays a vital role in regulating self-renewal of normal and cancer stem cells. BMI1 stimulates the self-renewal of normal and leukaemic stem cells (Lessard and Sauvageau, 2003), and enhances self-renewal of hematopoietic stem cells (Iwama et al., 2004). Bmi1 promotes neural stem self-renewal by repressing the p16 and p19 senescence pathways (Lessard and Sauvageau, 2003), but other studies have shown BMI1 controls neural stem self-renewal through p21-Rb pathway (Christopher A. Fasano et al., 2007). BMI1 is a regulator of prostate stem cell self-renewal and malignant transformation (Lukacs et al., 2010). BMI1 is a marker for intestinal stem cells, and a BMI1 inhibitor has been found to inhibit colorectal cancer stem cells (Kreso et al., 2014; Yan et al., 2012). In BCSCs, BMI1 is a target of miR-200 family and miR-128, which regulate BCSCs by targeting BMI1. BMI1 is also regulated by some signalings, such as Hedgehog (Liu et al., 2006). Activation of Hedgehog signaling pathway promotes self-renewal in both mammary stem/progenitor cells and BCSCs, and downregulation of BMI1 eliminates this effect. BMI1 also activates Wnt signaling pathway by repressing WNT inhibitors, Dickkopf (DKK), to activate WNT pathway. BMI1 also auto-activates itself. c-Myc is a target of WNT, and also an activator of BMI1. BMI1, DKK1, WNT and c-Myc form a positive feedback loop to promote BCSCs (Cho et al., 2013). So BMI1 plays a critical role in self-renewal of BCSCs (Fig. 2). Recently, Xu and colleagues reported that a lncRNA *FAL1* is overexpressed in some types of cancers including breast cancer, and it interacts with BMI1-PRC1 complex to enhance its stabilization by blocking its proteasomal degradation, which influences the ubiquitylation levels of H2AK119 to epigenetically repress genes expression such as CDKN1A (Neven et al., 2014). PRC1 binds to the target sites through PREs, and PREs may be ncRNAs or proteins, such as Jarid2, KDM2B and HOTAIR (Schwartz and Pirrotta, 2013). These suggest BMI1 can cross-talk with ncRNAs.

Histone-modifiers are a new frontier for drug discovery, and lots of small molecular compound inhibitors have been developed for disease therapies, among which some have been investigated in Phase III of clinical trials (Arrowsmith et al., 2012). The research of histone-modifiers in depth in regulating BCSCs will benefit the survival rate of patients with breast cancer and other type cancers.

## CONCLUDING REMARKS

In this review, we mainly summarize the role of ncRNA and histone-modifiers in regulating BCSCs. More and more non-coding RNAs have been been found to regulate CSCs, and the new function and mechanism of histone-modifiers is also getting clearer.

miRNAs are ncRNAs with short sequence, and they can be covalently conjugated with nanovectors easily. The tumor cells will be eradicated when the miRNAs or antisense nucleotide of specific miRNAs can be delivered to tumor cells by nanovectors. So, the development of nanocarriers of the drugs is important. lncRNAs are newly identified regulators of CSCs, and the regulation mechanisms are getting clearer by investigating more and more lncRNAs. lncRNAs can also function as therapeutic targets. For example, inhibiting lncRNA *BCAR4* with locked nucleic acid (LNA)-based antisense oligonucleotides suppressed the metastasis of breast cancer (Xing et al., 2014).

CSCs could not be cultured like embryonic stem cells in undifferentiated state *in vitro* to date, and flow cytometry with specific markers is the main method for CSC separation. Some important technologies used to study mechanism, such as immunoprecipitation and ChIP-seq, require a larger number of cells, which is not suitable for cancer stem cell research. But new technologies such as single cell RNA-sequencing (Hou et al., 2012) and ChIP-seq of 500 cells (Lara-Astiaso et al., 2014), will bring new hope for CSC research. For example, epigenetic regulator proteins play an important role in the regulation of CSCs, but the mechanism of regulation has still not been well studied due to the small CSCs number, single cell RNA-sequencing and ChIP-seq of 500 cells will eventually solve this problem. The promises and challenges of single cell RNA-sequencing have been reviewed by Stegle, O. and colleagues (Stegle et al., 2015).

The interaction among signaling pathways, ncRNAs and histone-modifiers plays a vital role in regulating CSCs. They can cross-talk with each other. Fully understanding the network of signaling pathways, ncRNAs and histone-modifiers will be helpful for tumor therapies.
